# Effects of tillage and biochar on soil physiochemical and microbial properties and its linkage with crop yield

**DOI:** 10.3389/fmicb.2022.929725

**Published:** 2022-09-20

**Authors:** Wenju Chen, Peipei Li, Fang Li, Jingjing Xi, Yanlai Han

**Affiliations:** ^1^College of Resources and Environmental Sciences, Henan Agricultural University, Zhengzhou, China; ^2^College of Life Sciences, Henan Agricultural University, Zhengzhou, China

**Keywords:** deep tillage, biochar, clayey soil, physicochemical properties, bacterial community structure, redundancy analysis, structural equation modeling

## Abstract

Vertisols are clayey soils with a high potential for improving production. Therefore, understanding the impact of tillage and fertilization on soil physicochemical properties and microbial community is essential for improving the vertisols with a high montmorillonite and smectite clay content. A 3-year field experiment was conducted to compare the effects of different tillage and fertilization practices at three depths of the vertisol under the wheat–maize cropping system in the North China Plain. The experimental treatments included rotary tillage without fertilization (R-CK), rotary tillage with chemical nitrogen (N), phosphorus (P), and potassium (K) fertilization (R-NPK), R-NPK plus biochar (R-NPKB), deep tillage without fertilization (D-CK), deep tillage with chemical N, P, and K fertilization (D-NPK), and D-NPK plus biochar (D-NPKB). The results showed that D-NPKB significantly improved winter wheat and summer maize yields by 14.4 and 3.8%, respectively, compared with R-NPK. The nitrate (NO_3_^–^–N) content of the deeper soil layer in D-NPKB was significantly higher than that in D-NPK. Meanwhile, biochar application increased the pH in the three layers. Compared with R-NPK, D-NPKB significantly increased the average content of available phosphorus (AP), soil organic carbon (SOC), and total nitrogen (TN) by 73.7, 18.5, and 19.0%, respectively. Meanwhile, *Gaiellale*, *Sphingomonadaceae*, and *Nocardioidaceae* were the predominant bacteria at the family level across all treatments, with a total relative proportion ranging from 14.1 to 23.6%. In addition, the abundance of *Bacillaceae* in deep tillage was 9.4% higher in the 20–30-cm soil layer than that in rotary tillage. Furthermore, the correlation analysis revealed a significant positive correlation between crop yield and chemical factors such as NO_3_^–^–N and the abundances of *Gaiellalea*, *Sphingomonadaceae*, and *Nocardioidaceae*. The findings collectively indicated that deep tillage combined with biochar application could increase the soil nutrients and modify the bacterial structure in the deeper soil layer and therefore will be beneficial for improving the productivity of the vertisols.

## Introduction

A vertisol is a heavy clay soil dominated by smectite minerals, especially montmorillonite (Wu et al., 2015); it is characterized by high soil bulk density and strength, low water permeability, and significant shrinkage and swelling capacity (Novelli et al., 2020; Xiong et al., 2020). It is the most widely distributed and important agricultural soil, mainly in Australia, India, Africa, and China (Ahmad, 1996). In China, the vertisol, located in the southern part of the North China Plain with approximately 4 million hectares, is one of the main soil types for low crop production. Additionally, long-term rotary tillage further leads to a shallow plowing layer and a thick plow pan, which restricts crop growth and yield in this soil (Wang et al., 2021). The vertisols have a high productivity potential due to a high montmorillonite and smectite clay fraction (DeCarlo and Caylor, 2020). And, therefore we need to explore a measure to overcome the physical barriers and nutrition limits in deeper soil, so as to improve crop yield and help in sustainable soil development. Therefore, it is necessary to improve the physical structure and chemical properties of the vertisols.

Tillage is a widely recognized effective practice for improving soil physical quality and crop yield (Yu et al., 2020; Wang et al., 2021). Previous studies have shown that reasonable tillage methods, such as subsoiling, deep plowing, and deep mixing of soil profile combined with rotary cultivation, improve soil fertility and slow down soil degradation and are conducive to a virtuous cycle of farmland ecosystems and efficiency (Aziz et al., 2013; Wang et al., 2020; Lopes et al., 2021). Moreover, mechanical soil profile modifications, commonly called deep tillage, can alleviate subsoil strength and facilitate deeper plant rooting (Schneider et al., 2017). Deep tillage could effectively lower the soil bulk density and improve crop yield and soil properties, especially the soil organic carbon (SOC) content, in the agricultural fields (Feng et al., 2020; Hu et al., 2021; Wang et al., 2021). Biochar is an important soil amendment, with a large specific surface area, well-developed pore structure, strong adsorption capacity, and high nutrient content, and is effective in increasing soil pH in acid soils (Obia et al., 2015; Hussain et al., 2020; Pan et al., 2021). Recent studies have shown that biochar application significantly improved soil fertility and changed the soil microbial structure and diversity (Liu et al., 2017; Xu et al., 2020).

Tillage and biochar play an important role in agricultural production. These methods directly affect the soil microbes *via* the input of nutrients and indirectly by altering the soil properties (Abujabhah et al., 2016; Chen et al., 2021). Recent studies have suggested that deep tillage and biochar application significantly reduced the bulk density of the vertisols and increased the soil air capacity in the topsoil layer and the content of SOC and available phosphorus (AP), subsequently improving crop yield (Fang et al., 2019; Liu et al., 2021; Wang et al., 2021). Therefore, deep tillage combined with biochar application can be a potential measure to overcome the shortcomings of shallow plowing layers and poor nutrient supply, thereby improving clay vertisols. These studies on improving clay soils with poor soil structure and shallow plowing layers mostly focused on improving the mixed soil layer (Chen et al., 2020; Lopes et al., 2021; Wang et al., 2021). However, the comparison study of physicochemical and microbial properties of different soil layers along the depth of different tillage systems has not been characterized. Besides, knowledge on improving the soil physicochemical properties and bacterial community of the separated soil layers is poor.

In this study, a consecutive field comparative experiment was conducted with contrasting tillage and fertilization practices carried out on a vertisol soil of Southern Henan Province, North China. This study aimed to investigate the effects of tillage and biochar on the soil physicochemical properties, microbial structure along various soil depths, and Spearman’s correlations among crop yield, soil physicochemical properties, and the bacterial community structure. We hypothesized that deep tillage combined with biochar application could improve soil physicochemical properties and microbial diversity of the deeper soil layer, subsequently enhancing crop yield. The study will provide a valuable theoretical basis for planning fertilization and tillage practices for the sustainable development of the vertisols.

## Study area

The experiment was conducted in a field in Baimiao Village, Xiping County, Henan Province, China (33°25′39″N, 113°49′23″E). The experimental area had a humid continental monsoon climate with four distinct seasons, with a mean annual precipitation of 852 mm, an air temperature of 14.8°C, and an average yearly frost-free period of 221 days. The primary cropping system of this area included summer maize (June–October) and winter wheat (October–next June). The soil type of the experimental area was classified as vertisol (FAO-UNESCO-ISRIC, 1988), with the contents of clay 50%, silt 37%, and sand 13% and with the following soil profile structure characteristics: A surface layer (0–20 cm) was heavy loam or light clay, with a granular structure and no calcareous nodules; a core layer (30–50 cm) was heavy soil or light sticky and a few are heavy sticky, with a fragmentary structure and a lot of ferromanganese nodules; and a bottom layer (>50 cm) was light sticky or heavy clay, with a block structure and a lot of calcareous nodules. The soil properties (0–20 cm) at the start of the experiment were as follows: pH 5.78; SOC 10.81 g kg^–1^; total soil nitrogen (TN) 1.26 g kg^–1^; AP 19.46 mg kg^–1^; and available potassium (AK) 171.6 mg kg^–1^. In this region, the field had been mainly cultivated by rotary tillage with 20 cm depth, which was by a four-wheel tractor with a hydraulic system.

## Materials and methods

### Experimental design

The tillage and fertilization trial was established in 2018 using a split-plot design with six blocks, including tillage as the main plot and fertilization as the split plot. Six treatments were carried out in the experimental plots as follows: (1) rotary tillage without fertilization (R-CK); (2) rotary tillage with chemical N, P, and K fertilization (R-NPK); (3) R-NPK plus biochar (R-NPKB); (4) deep tillage without fertilization (D-CK), (5) deep tillage with chemical N, P, and K fertilization (D-NPK); and (6) D-NPK plus biochar (D-NPKB). Each treatment was designed with three replicates; the area of each plot was 36 m^2^ (6 m × 6 m), with neighboring plots separated by a buffer strip of about 1 m. The local fertilization, with pure N, 180 kg hm^–2^, P_2_O_5_, 90 kg hm^–2^, and K_2_O, 90 kg hm^–2^ per season, was carried out in all treatments, except R-CK and D-CK. The P and K fertilizers were applied at the time of sowing as basal dressing, while 60% of N was applied as basal fertilizer and the remaining 40% as topdressing at the wheat jointing period and the maize big trumpet period, respectively. Maize straw biochar with a C/N ratio of 58.0 and a pH of 8.3 was obtained *via* pyrolysis at 500°C under anaerobic conditions from Hubei Jinri Eco-Energy Co., Ltd. (Anlu City, China). The biochar was applied at a 7500 kg ha^–1^ rate into the soil by an adjustable rotary tiller at 0–15 cm depth and 0–30 cm depth before sowing winter wheat. In the tillage system, a four-wheel tractor was used to pull the rotary tiller, with the operating depth adjusted using the tractor hydraulic system. And the burial depths of the rotary and deep tillage were 20 and 30 cm, respectively. A C-type blade attached to the rotary tiller destructed the deeper soil structure, especially in the deep tillage system; it broke the obstacles and promoted the intensive mixing of subsoil and topsoil. Moreover, the two wheels at the back of the rotary tiller were used to maintain the same operating depth. Following the local tillage regimes, summer maize seeds were directly sown after wheat harvest without any tillage.

### Grain yield and soil sampling

We collected three winter wheat plots of 1 m^2^ per treatment to determine the wheat grain yield under different treatments. Meanwhile, to determine the summer maize yield, we harvested 18 plants from the middle rows and acquired the seeds by a corn thresher, weighed the seeds after drying at 75°C, and calculated the yield for the total plants per hectare.

Soil samples were collected from three soil layers (0–10, 10–20, and 20–30 cm) from three replicate plots of each treatment after wheat and maize harvest in 2019 and 2020 and at the maize filling stage in 2020. Five soil samples were randomly collected from each plot, placed in labeled plastic bags, and immediately taken to the laboratory for further processing. In total, 252 samples from five periods and three soil layers were collected for physicochemical property analysis and 45 samples from three soil layers at the maize filling stage in 2020 for bacterial community analysis. The soil samples were sieved through a 2-mm sieve and divided into two subsamples: They were stored at 4°C for NH_4_^+^/NO_3_^–^–N (Li et al., 2020) and air-dried for chemical analysis. In particular, we additionally separated a part of soil samples from three soil layers collected at the maize filling stage in 2020 and stored at −80°C for soil microbial and molecular analysis.

### Soil physicochemical properties

The soil moisture was measured by oven-drying the samples at 105°C to a constant mass. The soil ammonium nitrogen (NH_4_^+^–N) and nitrate (NO_3_^–^–N) were extracted from the moist field soil stored at 4°C with 2 M KCL and estimated with a flow injection analyzer (SAN++, Sklar, Promega, Netherlands). The air-dried soil samples were sieved through a 100-mesh sieve and used to analyze the TN and SOC. The soil pH was determined at a soil-to-water ratio of 1:2.5 using a pH meter (Sartorius, PB-10), and SOC was determined following the classical potassium dichromate oxidation–ferrous sulfate titration method (Nelson and Sommers, 1982). Meanwhile, the soil samples were boiled with concentrated hydrogen peroxide and sulfuric acid, and the TN was determined by the Kjeldahl method (Kjeldahl, 1883). The AP was extracted using 0.5 M NaHCO_3_ and measured using the Olsen method (Olsen et al., 1954). The soil AK was extracted using 1 M NH_4_OAc and analyzed by flame emission spectrometry (Bao, 2000).

### DNA extraction and PCR amplification

Microbial genomic DNA was extracted from the soil samples using the FastDNA^®^ Spin Kit for Soil (MP Biomedicals, Santa Ana, CA, USA) according to the manufacturer’s instructions. The DNA extract was assessed on 1% agarose gel, and the DNA concentration and purity were determined with a NanoDrop 2000 UV–Vis spectrophotometer (Thermo Scientific, Wilmington, NC, USA). The hypervariable regions V3–V4 of the bacterial 16S rRNA gene were amplified using the primer pairs 338F (5′-ACTCCTACGGGAGGCAGCAG-3′) and 806R (5′-GGACTACHVGGGTWTCTAAT-3′) (Xu et al., 2016) on an ABI GeneAmp^®^ 9700 PCR thermocycler (ABI, Los Angeles, CA, USA). The PCR amplification of the 16S rRNA gene was performed as follows: initial denaturation at 95°C for 3 min, followed by 27 cycles of denaturing at 95°C for 30 s, annealing at 55°C for 30 s, and extension at 72°C for 45 s, and final extension at 72°C for 10 min. The PCR mixture contained 4 μL of 5 × TransStart FastPfu buffer, 2 μL of 2.5 mM dNTPs, 0.8 μL of each of the forward (5 μM) and reverse primers (5 μM), 0.4 μL of TransStart FastPfu DNA polymerase, and 10 ng of template DNA, made up to a final volume of 20 μL with ddH2O. PCR of each sample was performed in triplicate. The PCR product was purified from 2% agarose gel using the AxyPrep DNA Gel Extraction Kit (Axygen Biosciences, Union City, CA, USA) according to the manufacturer’s instructions and quantified using a Quantus™ fluorometer (Promega, USA).

### Illumina MiSeq sequencing

The purified amplicons were pooled in equimolar concentrations and paired-end sequenced (2 × 300) on an Illumina MiSeq platform (Illumina, San Diego, CA, USA) according to the standard protocols by Majorbio Bio-Pharm Technology Co., Ltd. (Shanghai, China). The raw reads were deposited into the NCBI Sequence Read Archive (SRA) database.

### Processing of sequencing data

The raw 16S rRNA gene sequencing reads were demultiplexed, quality-filtered by Fastp (version 0.19.6), and merged using FLASH (version 1.2.7) with the following criteria: (i) 300-bp reads were truncated at any site receiving an average quality score of <20 over a 50-bp sliding window, the truncated reads shorter than 50 bp were discarded, and the reads containing ambiguous characters were also discarded; (ii) only overlapping sequences longer than 10 bp were assembled according to their overlapped sequence. The maximum mismatch ratio of the overlap region allowed was 0.2. The reads that could not be assembled were discarded; and (iii) the samples were distinguished according to the barcode and primers, and the sequence direction was adjusted, with exact barcode matching and two nucleotide mismatches in primer matching.

Operational taxonomic units (OTUs) at a 97% similarity cutoff (Tipton et al., 2021) were clustered using UPARSE (version 7.1),^1^ and the chimeric sequences were identified and removed. The taxonomy of each OTU representative sequence was analyzed by RDP Classifier^2^ against the 16S rDNA database (Silva SSU128) using a confidence threshold of 0.7 (Wang et al., 2007).

### Data analysis

Data were represented as mean ± standard error for all the assessed parameters. Statistical analysis was performed using Excel 2016 and SPSS 23.0 software (IBM Co., Armonk, NY, USA). The differences in crop yield, soil moisture, NH_4_^+^–N, NO_3_^–^–N, pH, SOC, TN, AP, and AK between the different treatments at the same depth were analyzed using one-way analysis of variance (ANOVA) and Duncan’s multiple range test (DMRT) at a 5% significance level. Repeated measure analyses of variance (RMANOVA) with crop yield and soil properties (physicochemical properties and bacterial alpha diversity at the genus level) were performed to probe the effect of tillage, fertilization, and season. Interaction (tillage × fertilization, tillage × season, fertilization × season, and tillage × fertilization × season) and main effect (tillage, fertilization, and season) were tested. The vegan package in R language software (version 3.5.1) was used to conduct a redundancy analysis (RDA) based on the microbe and the soil physicochemical properties. Besides, the structural equation modeling (SEM) was performed to examine the direct and indirect effects of soil physicochemical properties on the bacterial richness and yield using IBM SPSS Statistics (version 23) and IBM SPSS Amos Graphics (version 21.0.0) (Build 1178) (*P* < 0.05). We selected bacteria at the family level with a mean relative abundance greater than 0.05% within each soil layer and OTUs with a relative abundance greater than 0.05% for Spearman’s correlation analysis. Then, the taxon–taxon co-existing network and the taxon–environment network were constructed to examine the association between various taxa and between taxa and the environment within the three soil layers using the R language software (version 4.1.0) with “psych” package and Gephi (version 0.9.2).

## Results

### Soil physicochemical analysis

#### Effect of tillage and biochar on crop yield

The trends of wheat and maize yields to tillage and biochar treatments for three consecutive years from 2018 to 2020 are given in Table 1. Varying degrees of differences in crop yields were observed among the different tillage and fertilization treatments. In the first crop year (2018), the winter wheat and summer maize yields in the R-NPKB treatment significantly increased by 8.5 and 9.2%, respectively, compared with those in the R-NPK treatment (*P* < 0.05). Meanwhile, the wheat and maize yields in the D-NPKB treatment in 2019 significantly increased by 8.4 and 8.9%, respectively, compared with those in the D-NPK treatment and by 9.9 and 26.8%, respectively, compared with those in the R-NPK treatment. The summer maize yield in the R-NPKB treatment was 10,635 kg ha^–1^, higher than in the R-NPK treatment in 2018. Deep tillage significantly increased the summer maize yield (5,792 kg ha^–1^) in the NPKB treatment compared with the yield under rotary tillage (4,538 kg ha^–1^) following the same fertilization in 2019. Thus, the average winter wheat and summer maize yields in the 3 years were 14.4 and 3.8%, respectively, higher in the D-NPKB treatment than those in the R-NPK treatment. In general, deep tillage combined with biochar application increased the crop yield compared with the other treatments in the 3 years, except for the summer maize in 2018. The analysis of the interaction revealed that deep tillage significantly increased the maize yield (*P* < 0.01), while the impact on wheat yield was significant only in 2020. Besides, fertilization and biochar application significantly improved the winter wheat and summer maize yields compared with the CK treatments (*P* < 0.001).

**TABLE 1:** Responses of the grain yield (kg ha^–1^) to different tillage and fertilization practices in the wheat–maize rotation system throughout 2018–2020 cropping years.

Tillage	Treatment	2018 wheat	2018 maize	2019 wheat	2019 maize	2020 wheat	2020 maize
R	CK	6033 ± 115^de^	9401 ± 224^b^	3395 ± 289^c^	3390 ± 291^c^	2570 ± 155^d^	7633 ± 195^d^
	NPK	6330 ± 407^cd^	9740 ± 563^b^	8564 ± 255^b^	4568 ± 301^b^	7067 ± 696^bc^	8827 ± 182^a^
	NPKB	6871 ± 169^b^	10635 ± 192^a^	9231 ± 92^a^	4538 ± 251^b^	6593 ± 219^c^	8575 ± 110b
D	CK	5702 ± 247e	8687 ± 68^c^	3489 ± 313^c^	3546 ± 396^c^	2810 ± 212^d^	7977 ± 174c
	NPK	6729 ± 179^bc^	9248 ± 222^bc^	8684 ± 428^b^	5319 ± 253^a^	7540 ± 470^ab^	8939 ± 96^a^
	NPKB	7581 ± 325^a^	9258 ± 533^bc^	9415 ± 330^a^	5792 ± 472^a^	8133 ± 180^a^	8965 ± 67^a^
Tillage (T)		4.417	27.002***	0.863	20.562**	18.563**	19.332**
Fertilization (F)		40.836***	9.891**	679.169***	44.923***	315.329***	114.031***
T × F		6.315*	2.573	0.035	3.988*	5.268*	0.206

The letters a, b, c, d, and e in a column indicate significant differences among different tillage and treatments at the level of *P* < 0.05. Values of R and D are mean ± standard deviation, the values of T, F, and T × F in the table represent *F*-value, and significance levels are denoted with *P < 0.05, **P < 0.01, and ***P < 0.001. R, rotary tillage; D, deep tillage; CK, no fertilization; NPK, chemical nitrogen (N), phosphorus (P), and potassium (K) fertilization; NPKB, NPK plus biochar.

#### Effect of tillage and biochar on soil physical properties

Table 2 shows the bulk density of soil at different depths during the winter wheat and summer maize seasons in 2019 and maize in 2020. The NPK treatment significantly increased the average soil bulk density by 4.9% compared with the CK treatment, while the NPKB treatment significantly decreased by 4.2% compared with the NPK treatment in the 0–10- and 10–20-cm soil layers during the wheat and maize seasons in 2019. Meanwhile, the NPKB treatment lowered the soil bulk density (*P* < 0.01) in the 0–10-cm and 20–30-cm soil layers compared with the NPK treatment for most crop seasons. Under the same fertilization, deep tillage had the soil bulk density values of 1.51 g cm^–3^ (D-CK and D-NPKB) and 1.57 g cm^–3^ (D-CK) in the 10–20- and 20–30-cm soil layers for the 2019 summer maize. Besides, soil bulk density significantly increased with the treatment time in the 0–10-cm soil layer and significantly decreased in the 20–30-cm soil layer (*P* < 0.001) from 2019 to 2020. Interactive analysis revealed that deep tillage significantly lowered the soil bulk density in the 10–20- and 20–30-cm soil layers (*P* < 0.01) compared with rotary tillage, but no significant change was detected in the 0–10-cm soil layer.

**TABLE 2:** Responses of the soil bulk density (g cm^–3^) to different tillage and fertilization practices in the wheat–maize rotation system throughout 2019–2020 cropping years.

Season	Tillage	Treatment	0–10 cm	10–20 cm	20–30 cm
The mature stage of wheat in 2019	R	CK	1.19 ± 0.04^bc^	1.50 ± 0.03^b^	1.65 ± 0.01^ab^
		NPK	1.26 ± 0.05^a^	1.63 ± 0.05^a^	1.67 ± 0.02^a^
		NPKB	1.19 ± 0.02^bc^	1.49 ± 0.01^b^	1.67 ± 0.06^a^
	D	CK	1.18 ± 0.01^c^	1.50 ± 0.05^b^	1.57 ± 0.02^c^
		NPK	1.24 ± 0.01^ab^	1.55 ± 0.02^ab^	1.64 ± 0.04^ab^
		NPKB	1.19 ± 0.01^c^	1.53 ± 0.10^ab^	1.59 ± 0.03^bc^
The mature stage of maize in 2019	R	CK	1.29 ± 0.03^b^	1.52 ± 0.02^ab^	1.69 ± 0.02^a^
		NPK	1.36 ± 0.04^a^	1.57 ± 0.03^a^	1.69 ± 0.01^a^
		NPKB	1.31 ± 0.03^ab^	1.52 ± 0.02^ab^	1.69 ± 0.03^a^
	D	CK	1.30 ± 0.01^b^	1.51 ± 0.02^c^	1.57 ± 0.04^c^
		NPK	1.37 ± 0.02^a^	1.55 ± 0.04^ab^	1.63 ± 0.01^b^
		NPKB	1.31 ± 0.05^ab^	1.51 ± 0.04^c^	1.59 ± 0.01^bc^
The mature stage of maize in 2020	R	CK	1.48 ± 0.07^a^	1.58 ± 0.02^a^	1.52 ± 0.06^b^
		NPK	1.48 ± 0.08^a^	1.59 ± 0.03^a^	1.62 ± 0.03^a^
		NPKB	1.42 ± 0.11^a^	1.61 ± 0.05^a^	1.58 ± 0.03^ab^
	D	NPK	1.44 ± 0.10^a^	1.48 ± 0.06^b^	1.59 ± 0.07^ab^
		NPKB	1.41 ± 0.03^a^	1.53 ± 0.05^ab^	1.60 ± 0.02^ab^
Season (S)			76.066***	0.599	17.910***
Tillage (T)			0.530	8.509**	31.948***
Fertilization (F)			5.509**	4.582*	9.146**
S × T			0.301	3.153	3.739*
S × F			0.686	2.790*	1.453
T × F			0.073	1.955	0.892
S × T × F			0.043	0.757	1.079

The letters a, b, and c in a column indicate significant differences among different tillage and treatments at the level of *P* < 0.05. Values of R and D are mean ± standard deviation, and the values of S, T, F, S × T, S × F, T × F, and S × T × F in the table represent *F*-value, and significance levels are denoted with *P < 0.05, **P < 0.01, and ***P < 0.001. R, rotary tillage; D, deep tillage; CK, no fertilization; NPK, chemical N, P, and K fertilization; NPKB, NPK plus biochar. The same below.

The soil moisture under different treatments from 2019 to 2020 is given in Table 3. In our research, the soil moisture in the summer maize season increased from the 0–10-cm soil layer to the 20–30-cm soil layer in 2019, while it decreased along with the soil profile of each treatment in 2020. The soil moisture significantly differed among the seasons (*P* < 0.05), with no significant differences between treatments in 2019.

**TABLE 3:** Responses of the soil moisture content (%) to different tillage and fertilization practices in the wheat–maize rotation system in 2019–2020.

Season	Tillage	Treatment	0–10 cm	10–20 cm	20–30 cm
The mature stage of wheat in 2019	R	CK	4.53 ± 0.89^b^	17.84 ± 1.73^a^	15.93 ± 0.84^abc^
		NPK	6.61 ± 0.86^ab^	19.28 ± 1.36^a^	14.87 ± 0.78^abc^
		NPKB	6.65 ± 0.75^ab^	19.91 ± 1.29^a^	14.30 ± 0.74^bc^
	D	CK	6.65 ± 0.76^ab^	19.19 ± 1.01^a^	16.14 ± 1.13^ab^
		NPK	6.65 ± 0.77^a^	19.18 ± 1.05^a^	13.96 ± 1.34^c^
		NPKB	6.65 ± 0.78^ab^	19.16 ± 0.30^a^	16.58 ± 1.56^a^
The mature stage of maize in 2019	R	CK	19.85 ± 2.42^a^	22.37 ± 0.78^a^	21.06 ± 2.19^a^
		NPK	20.24 ± 0.44^a^	21.64 ± 1.20^a^	19.63 ± 0.13^a^
		NPKB	19.29 ± 2.07^a^	21.48 ± 1.55^a^	19.70 ± 0.66^a^
	D	CK	20.28 ± 1.86^a^	21.02 ± 0.32^a^	19.49 ± 0.25^a^
		NPK	20.06 ± 2.43^a^	20.90 ± 0.34^a^	20.00 ± 0.78^a^
		NPKB	20.70 ± 0.82^a^	21.60 ± 1.28^a^	19.49 ± 0.36^a^
The mature stage of wheat in 2020	R	CK	5.19 ± 0.89^ab^	7.51 ± 1.24^b^	9.26 ± 0.33^c^
		NPK	6.47 ± 1.55^ab^	8.63 ± 0.96^b^	9.23 ± 0.64^c^
		NPKB	5.67 ± 1.80^ab^	9.20 ± 1.12^b^	10.64 ± 1.35^bc^
	D	CK	4.71 ± 0.44^b^	8.52 ± 0.37^b^	10.48 ± 0.31^bc^
		NPK	5.91 ± 0.21^ab^	12.47 ± 1.00^a^	14.14 ± 2.53^a^
		NPKB	7.48 ± 1.41^a^	11.44 ± 1.58^a^	12.49 ± 1.50^ab^
The filling stage of maize in 2020	R	CK	21.60 ± 0.38^b^	20.23 ± 0.42^b^	19.17 ± 0.11^b^
		NPK	22.83 ± 0.45^a^	21.36 ± 0.33^ab^	20.15 ± 0.48^ab^
		NPKB	22.84 ± 0.50^a^	21.04 ± 0.52^ab^	19.81 ± 0.64^ab^
	D	NPK	22.93 ± 0.42^a^	21.62 ± 1.18^a^	19.78 ± 0.80^ab^
		NPKB	22.93 ± 0.43^b^	21.50 ± 0.28^a^	20.48 ± 0.70^a^
The mature stage of maize in 2020	R	CK	21.59 ± 0.78^a^	19.17 ± 1.01^ab^	19.50 ± 0.73^a^
		NPK	22.15 ± 0.80^a^	19.69 ± 0.87^ab^	18.97 ± 0.37^a^
		NPKB	21.53 ± 0.51^a^	18.11 ± 1.76^b^	18.34 ± 0.54^a^
	D	NPK	22.05 ± 1.84^a^	21.11 ± 0.04^a^	19.19 ± 1.25^a^
		NPKB	21.06 ± 1.21^a^	20.86 ± 1.66^a^	19.19 ± 1.31^a^
S			709.338***	368.910***	232.051***
T			0.085	10.428**	5.775*
F			4.788*	4.868*	0.307
S × T			0.437	6.04***	6.445***
S × F			0.808	2.189*	3.080**
T × F			0.172	0.256	3.028
S × T × F			0.934	1.770	2.741

The letters a, b, and c in a column indicate significant differences among different tillage and treatments at the level of *P* < 0.05. Values of R and D are mean ± standard deviation, and the values of S, T, F, S × T, S × F, T × F, and S × T × F in the table represent *F*-value, and significance levels are denoted with *P < 0.05, **P < 0.01, and ***P < 0.001. R, rotary tillage; D, deep tillage; CK, no fertilization; NPK, chemical N, P, and K fertilization; NPKB, NPK plus biochar.

#### Effect of tillage and biochar on soil pH, available phosphorus, available potassium, soil organic carbon, and total nitrogen

Soil chemical properties, including pH, AP, AK, SOC, and TN, from 2019 to 2020 are listed in Supplementary Table. In the 10–20- and 20–30-cm soil layers at the winter wheat mature stage in 2019, the soil pH in the D-NPKB treatment was higher than that in the D-NPK treatment, which was 5.9 and 7.36, respectively. During the summer maize mature stage in 2019, the soil pH values ranged from 5.12 to 5.18 in the NPK and NPKB treatments under deep tillage, significantly lower than that in the CK treatment (6.19) in the topsoil layer. The soil pH in the R-NPKB treatment increased by 1.3 and 4.3% in the 10–20- and 20–30-cm soil layers, respectively, during the winter wheat season in 2020 compared with that in the R-NPK treatment. Under the same fertilization, the pH values of NPK and NPKB treatments under deep tillage were 5.58 and 5.77, respectively, which were higher than the corresponding treatments under rotary tillage (5.21 and 5.47, respectively) in the 0–10-cm soil layer.

During the mature stages of wheat and maize in 2019–2020, the AP, AK, SOC, and TN in the NPKB treatment increased to varying degrees compared with those in the NPK treatment in the 10–20- and 20–30-cm soil layers. The NPKB treatment significantly increased the average content of soil AP, AK, SOC, and TN in the three soil layers at the summer maize mature stage in 2020 by 9.7, 11.4, 11.7, and 3.9%, respectively, compared with the NPK treatment. The soil SOC levels in the NPKB treatment were 13.78 and 14.16 g kg^–1^ under rotary and deep tillage, respectively, which were higher than those in the NPK treatment (12.09 and 12.38 g kg^–1^) in the 10–20-cm soil layer at the mature maize stage in 2020. Under the same fertilization and season, deep tillage increased soil AP, TN, AK, and SOC content in the 20–30-cm soil layer for most crop seasons, and significant changes were detected in AP and TN content in both wheat and maize seasons in 2019 (*P* < 0.05). The D-NPKB treatment significantly increased the average content of AP, SOC, and TN in the 20–30-cm soil layer by 73.7, 18.5, and 19.0%, respectively, compared with the R-NPK treatment in the 2 years.

The interactive analysis revealed season and fertilization as the significant factors affecting the soil pH, AP, SOC, and TN. Fertilization combined with biochar application increased the soil AK, SOC, and TN content in the 20–30-cm soil layer.

#### Effect of tillage and biochar on soil NH_4_^+^–N and NO_3_^–^–N

The values of the soil NH_4_^+^–N and NO_3_^–^–N contents in each soil layer during the summer maize filling and mature stages in 2020 are given in Table 4. No significant difference was detected in the soil NH_4_^+^–N at the same stage among the treatments (*P* > 0.05). The soil NO_3_^–^–N content in the 0–30-cm soil layer decreased along with the soil profile of each treatment. The soil NO_3_^–^–N content in the NPK and NPKB treatments was significantly higher than that in the CK treatment in the 0–10-cm soil layer. In the summer maize filling stage, the soil NO_3_^–^–N content in the D-NPKB treatment was significantly higher than that in the D-NPK treatment in the 10–20-cm soil layer (*P* < 0.05). The soil NO_3_^–^–N content in the D-NPKB treatment was 21.05 mg kg^–1^, significantly higher than that in the D-NPK treatment (14.11 mg kg^–1^) in the 10–20-cm soil layer. In the summer maize mature stage, the NO_3_^–^–N content in the NPK and NPKB treatments under deep tillage increased by 21.9 and 76.3%, respectively, compared with the corresponding treatments under rotary tillage in the 10–20-cm soil layer. The interactive analysis showed that soil NH_4_^+^–N and NO_3_^–^–N contents changed with the growth of summer maize. The soil NH_4_^+^–N content in the mature stage was significantly lower (*P* < 0.001) than that in the maize filling stage in each soil layer, while the soil NO_3_^–^–N content showed an opposite trend (*P* < 0.05) in the 0–10- and 10–20-cm soil layers. The D-NPKB treatment significantly increased the soil NO_3_^–^–N content by 49.2 and 41.9% in the 10–20- and 20–30-cm soil layers, respectively, compared with the D-NPK treatment in the summer maize filling stage.

**TABLE 4:** Responses of the contents of NH_4_^+^–N and NO_3_^–^–N (mg kg^–1^) to different tillage and fertilization practices in the wheat–maize rotation system during the maize stages in 2020.

Season	Tillage	Treatment	NH_4_^+^-N (mg kg^–1^)			NO_3_^–^-N (mg kg^–1^)		
			0–10 cm	10–20 cm	20–30 cm	0–10 cm	10–20 cm	20–30 cm
The filling stage of maize in 2020	R	CK	4.48 ± 1.00^b^	3.92 ± 0.39^a^	4.90 ± 0.96^a^	6.82 ± 1.32^b^	3.22 ± 0.39^c^	3.38 ± 0.85^c^
		NPK	6.77 ± 1.97^a^	4.21 ± 0.44^a^	4.30 ± 0.28^a^	20.85 ± 1.12^a^	13.60 ± 0.59^b^	10.10 ± 0.79^ab^
		NPKB	4.25 ± 0.25^b^	4.52 ± 0.71^a^	4.51 ± 0.47^a^	21.02 ± 2.99^a^	15.26 ± 3.16^b^	8.24 ± 1.27^b^
	D	NPK	4.26 ± 0.69^b^	4.12 ± 0.43^a^	3.91 ± 0.22^a^	17.60 ± 3.20^a^	14.11 ± 2.20^b^	9.67 ± 0.82^b^
		NPKB	4.62 ± 0.99^b^	4.45 ± 1.50^a^	4.19 ± 0.46^a^	20.46 ± 3.94^a^	21.05 ± 4.71a	13.72 ± 4.06a
The maturation stage of maize in 2020	R	CK	1.29 ± 0.14^a^	0.53 ± 0.07^a^	0.96 ± 0.22^a^	13.73 ± 0.89^b^	8.31 ± 4.13^b^	3.49 ± 2.82^b^
		NPK	1.56 ± 0.15^a^	0.60 ± 0.16^a^	1.47 ± 0.90^a^	33.32 ± 10.30^a^	17.37 ± 9.56^ab^	5.35 ± 1.02^ab^
		NPKB	1.49 ± 0.06^a^	1.40 ± 0.90^a^	0.75 ± 0.29^a^	29.67 ± 6.74^a^	14.07 ± 5.37^ab^	6.26 ± 1.82^ab^
	D	NPK	4.06 ± 1.47^a^	1.57 ± 0.55^a^	1.20 ± 0.30^a^	26.81 ± 7.21^a^	21.17 ± 7.31^ab^	8.38 ± 2.91^ab^
		NPKB	3.08 ± 0.50^a^	1.84 ± 1.35^a^	0.65 ± 0.17^a^	29.76 ± 5.60^a^	24.80 ± 10.27^a^	9.30 ± 4.43^a^
S			57.028***	103.166***	317.475***	26.216***	3.663	5.875*
T			2.059	0.947	1.685	1.782	4.878*	7.662*
F			3.938*	1.552	0.673	23.103***	5.644*	5.935**
S × T			21.036***	1.476	0.172	0.116	0.760	0.065
S × F			0.794	0.055	3.104	0.421	0.600	1.004
T × F			2.109	0.149	0.085	1.469	1.678	2.175
S × T × F			7.828*	0.179	0.013	0.261	0.031	2.165

The letters a, b, and c in a column indicate significant differences among different tillage and treatments at the level of *P* < 0.05. Values of R and D are mean ± standard deviation, and the values of S, T, F, S × T, S × F, T × F, and S × T × F in the table represent *F*-value, and significance levels are denoted with *P < 0.05, **P < 0.01, and ***P < 0.001. R, rotary tillage; D, deep tillage; CK, no fertilization; NPK, chemical N, P, and K fertilization; NPKB, NPK plus biochar.

### Composition of the microbial community at different soil depths

**FIGURE 1:**
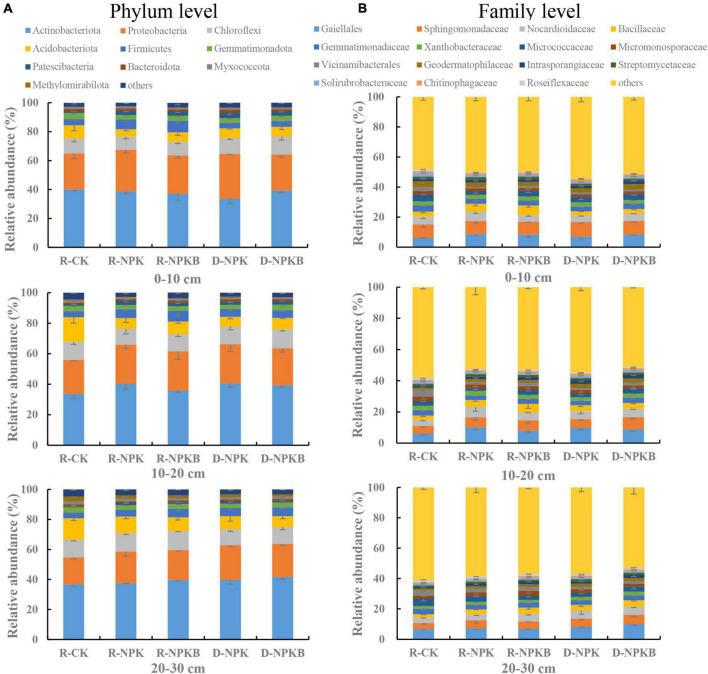
The differences in bacterial community composition among different treatments at the phylum and family levels in 0–10-cm, 10–20-cm, and 20–30-cm soil layers. **(A)** Actinobacteriota, proteobacteria, chloroflexi, acidobacteriota, firmicutes, gemmatimonadota, patescibacteria, bacteroidota, myxococcota, methylomirabilota, and others. **(B)** Gaiellales, sphingomonadaceae, nocardioidaceae, bacillaceae, gemmatimonadaceae, xanthobacteraceae, micrococcaceae, miceomonosporaceae, vicinamibacterales, geodermatophilaceae, intrasporangiaceae, streptomycelaceae, solirubrobacteraceae, chitinophagaceae, roseiflexaceae, and others. R-CK, rotary tillage without fertilization. R-NPK, rotary tillage with chemical nitrogen (N), phosphorus (P), and potassium (K) fertilization. R-NPKB, R-NPK plus biochar. D-NPK, deep tillage with chemical N, P, and K fertilization. D-NPKB, D-NPK plus biochar. The different colors represent the different bacterial species at the levels of phylum and family, which are arranged in descending order according to species abundance. The same below.

**FIGURE 2:**
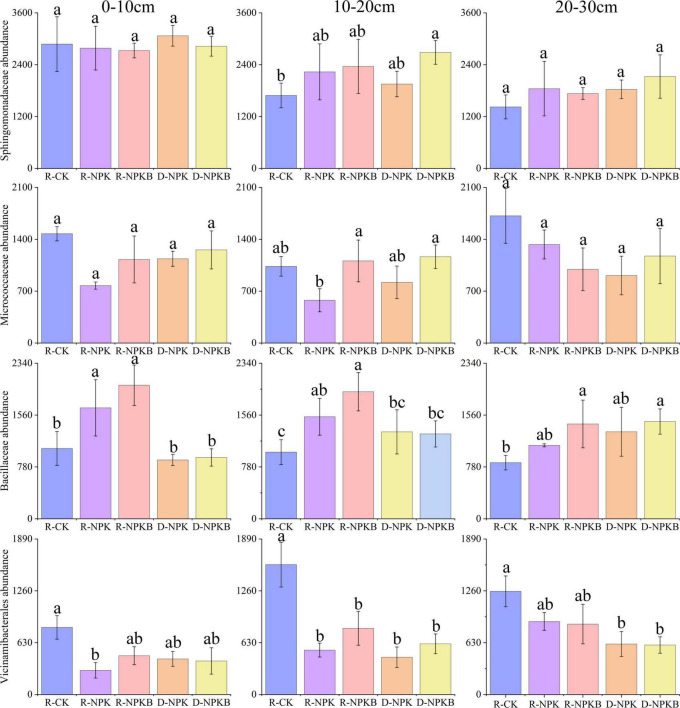
The differences in the abundances of *Sphingomonadaceae*, *Micrococcaceae*, *Bacillaceae*, and *Vicinamibacterales* at the family level among different treatments in the 0–10-cm, 10–20-cm, and 20–30-cm soil layers. The letters a, b, and c in each subgraph indicate significant differences among different tillage and treatments at the level of *P* < 0.05.

The top 10 bacterial phyla and 15 bacterial families with a relative abundance were selected to analyze the differences in the microbial composition under different treatments and at different soil layers (Figure 1). At the phylum level, the dominant bacterial phyla were *Actinobacteria*, *Proteobacteria*, *Chloroflexi*, and *Acidobacteria*, and the total relative abundance accounted for 79.6–84.5% (Figure 1A). The abundance of *Methylomirabilota* in the 20–30-cm soil layer was the highest in the three soil layers. The families *Gaiellale*, *Sphingomonadaceae*, and *Nocardioidaceae* were predominant under different treatments and at different soil layers with a total relative proportion ranging from 14.1 to 23.6% (Figure 1B). The *Sphingomonadaceae* abundance decreased with the increasing soil depth under each treatment, and the highest abundance in the 10–20- and 20–30-cm soil layers was under the D-NPKB treatment (Figure 2). The *Micrococcaceae* abundance under deep tillage increased by 25.8 and 17.7% in the 0–10- and 10–20-cm soil layers, respectively, compared with that under rotary tillage except for the R-CK treatment; however, the abundance lowered by 10.3% in the 20–30-cm soil layer. In the 0–10- and 10–20-cm soil layers, the *Bacillaceae* abundance under rotary tillage was higher than that under deep tillage except for the R-CK treatment, whereas an opposite trend was observed in the 20–30-cm soil layer. The *Bacillaceae* abundance in the 20–30-cm soil layer improved by 9.4% under deep tillage compared with that under rotary tillage except for the R-CK treatment, and the highest abundance was detected in D-NPKB treatment. Meanwhile, *Vicinamibacterales* abundance was higher in the R-CK treatment than that in the other treatments.

### Redundancy analysis of microbial community structure and soil properties

**FIGURE 3:**
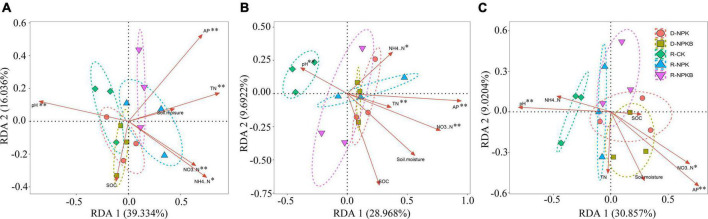
Redundancy analysis between soil physicochemical properties and the soil bacteria at the family level under different tillage methods and fertilizations in the 0–10-cm **(A)**, 10–20-cm **(B)**, and 20–30-cm **(C)** soil layers, and significant levels are denoted with *P < 0.05 and **P < 0.01. R-CK, rotary tillage without fertilization; R-NPK, rotary tillage with chemical N, P, and K fertilization; R-NPKB, R-NPK plus biochar; D-CK, deep tillage without fertilization; D-NPK, deep tillage with chemical N, P, and K fertilization; D-NPKB, D-NPK plus biochar; AP, available phosphorus; SOC, soil organic carbon; TN, total nitrogen; NH_4_^+^–N, ammonium nitrogen; NO_3_^–^–N, nitrate.

The differences in the correlations between soil physiochemical and bacterial community among the treatments were analyzed by RDA (Figure 3). The structure of bacterial communities at the family level showed obvious differences among different treatments; significant differences in bacterial community composition were detected between the tillage and fertilization treatments. In the 10–30-cm soil layers, the R-CK treatment appeared clearly separated from the other treatments (R-NPK, R-NPKB, D-NPK, and D-NPKB) (Figures 3B,C), revealing a clear difference in the bacterial community with tillage and biochar application. Besides, in the 10–20-cm soil layer, the R-NPKB and D-NPKB treatments appeared largely separated from the R-NPK and D-NPK treatments (Figure 3B), revealing a significant change in the bacterial community with biochar application. The total variation in the 0– 10-, 10– 20-, and 20–30-cm soil layers was 70.7, 66.7, and 55.4%, respectively, which reflected the influence of soil environmental factors on the structure of the soil bacterial community.

Further, a correlation analysis was performed to explore the effect of soil physicochemical properties on soil bacterial community (Figure 3). The soil pH, AP, and NO_3_^–^–N were positively related to the community structure in the three soil layers (*P* < 0.05), and soil NH_4_^+^–N and TN were positively related to the bacterial community in the 0–10-cm and 10–20-cm soil layers (*P* < 0.05). The soil moisture and SOC did not significantly affect the bacterial community in the three soil layers (*P* > 0.05).

### Alpha diversity indices of soil bacteria at the genus level

The bacterial diversity of 45 soil samples under rotary and deep tillage is given in Table 5. The coverage of all samples (coverage index) was over 99%, which indicated that the sequencing data were reliable and reflected the real situation of the soil bacterial community. Different tillage treatments significantly affected the evenness (ACE index; *P* < 0.05), but not the Shannon diversity index (*P* > 0.05) in the 0–10-cm soil layer. The ACE index in all treatments under deep tillage was higher than that under rotary tillage in the 0–10- and 10–20-cm soil layers. The richness (Chao’s index) based on the genus level in the NPK and NPKB treatments was significantly more than that in the CK treatment (*P* < 0.05) in the 0–10-cm soil layer. These findings indicate that the effect of different fertilization treatments was significant (*P* < 0.05) in the 0–10-cm soil layer. In the 20–30-cm soil layer, tillage significantly affected the Simpson index (*P* < 0.05); the Simpson index under deep tillage was higher than that under rotary tillage. However, no significant differences in alpha diversity were observed among the tillage and fertilization treatments in the 10–20-cm soil layer.

**TABLE 5:** Diversity indices of soil bacterial communities based at the genus level of different tillage and fertilization practices.

Sample	0–10 cm					10–20 cm					20–30 cm				
	Shannon	ACE	Chao	Simpson	Coverage	Shannon	ACE	Chao	Simpson	Coverage	Shannon	ACE	Chao	Simpson	Coverage
Rotary tillage															
CK	4.58^a^	593.71^c^	593.21^c^	0.0239^a^	0.9981^a^	4.73^a^	623.64^a^	631.29^b^	0.0179^b^	0.9977^a^	4.71^ab^	655.13^a^	656.59^a^	0.0190^b^	0.9977^a^
NPK	4.56^a^	633.76^ab^	645.78^ab^	0.0269^a^	0.9978^a^	4.59^a^	656.53^a^	657.60^ab^	0.0264^a^	0.9979^a^	4.77^a^	667.75^a^	665.15^a^	0.0188^b^	0.9978^a^
NPKB	4.59^a^	616.07^bc^	630.98^bc^	0.0259^a^	0.9976^a^	4.66^a^	686.73^a^	702.99^a^	0.0229^ab^	0.9974^a^	4.79^a^	681.37^a^	679.98^a^	0.0182^b^	0.9975^a^
Deep tillage															
NPK	4.68^a^	652.79^ab^	679.03^a^	0.0230^a^	0.9975^a^	4.69^a^	661.95^a^	663.62^ab^	0.0231^ab^	0.9979^a^	4.79^a^	667.08^a^	660.80^a^	0.0200^b^	0.9975^a^
NPKB	4.58^a^	660.90^a^	654.04^ab^	0.0253^a^	0.9979^a^	4.62^a^	648.82^a^	664.59^ab^	0.0240^ab^	0.9974^a^	4.65^b^	660.69^a^	675.63^a^	0.0246^a^	0.9974^a^
Two-way ANONA															
Tillage	0.335	**0.021**	0.064	0.271	0.839	0.694	0.469	0.432	0.655	0.859	0.075	0.356	0.784	**0.002**	0.333
Fertilization	0.820	0.137	**0.035**	0.548	0.504	0.472	0.226	0.113	0.088	0.118	0.054	0.374	0.469	0.122	0.601
T × F	0.246	0.297	0.715	0.401	0.261	0.295	0.339	0.288	0.352	0.927	**0.021**	0.386	1.000	**0.016**	0.539

0–10 cm, 10–20 cm and 20–30 cm in bold represent different soil layers. Shannon, ACE, Chao, Simpson and Coverage in bold represent Alpha diversity indexes. The letters a, b, and c in a column indicate significant differences among different tillage and treatments at the level of *P* < 0.05.

### Structural equation modeling analysis

**FIGURE 4:**
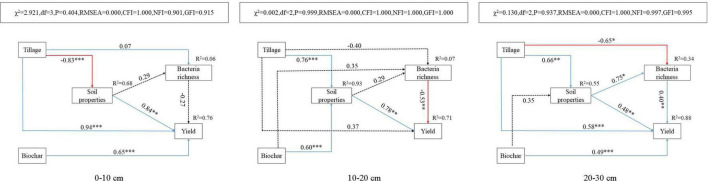
Analysis of factors affecting soil bacterial species richness with the structural equation modeling (SEM) in 0–10-cm, 10–20-cm, and 20–30-cm soil layers. The arrow and the number on the arrow represent the path coefficient, high or low; solid and dashed arrows represent the significant (*P < 0.05, **P < 0.01, and ***P < 0.001) and non-significant effects, respectively; and blue and red arrows represent the positive and negative correlation, respectively.

In this study, the SEM was set up to further clarify the effect of soil physicochemical properties on the richness of bacterial species and crop yield in the three layers of the vertisol (Figure 4). The model explained 6, 7, and 34% of the variation in soil bacterial species richness and 76, 71, and 88% of the variation in yield in the 0– 10-, 10– 20-, and 20–30-cm soil layers, respectively. Besides, the path coefficient was used in the model to estimate the magnitude of the effect of the independent variable on the corresponding dependent variable and compare its relative importance. Deep tillage significantly decreased the soil physicochemical properties of the 0–10-cm soil layer (*P* < 0.001), while it significantly improved the physicochemical properties of the 10–20-cm and 20–30-cm soil layers (*P* < 0.05). In addition, biochar application significantly improved the soil physicochemical properties of the 10–20-cm soil layer (*P* < 0.05). Deep tillage and biochar application directly or indirectly *via* affecting soil physicochemical properties significantly improved the crop yield (*P* < 0.05). Deep tillage had a significant negative effect on bacterial richness only in the 20–30-cm soil layer (*P* < 0.01), but showed no effect in the 0–10- and 10–20-cm soil layers (*P* > 0.05). Biochar application showed no effect on bacterial richness in the 10–20-cm soil layer (*P* > 0.05).

### Network analysis under various soil depths

**FIGURE 5:**
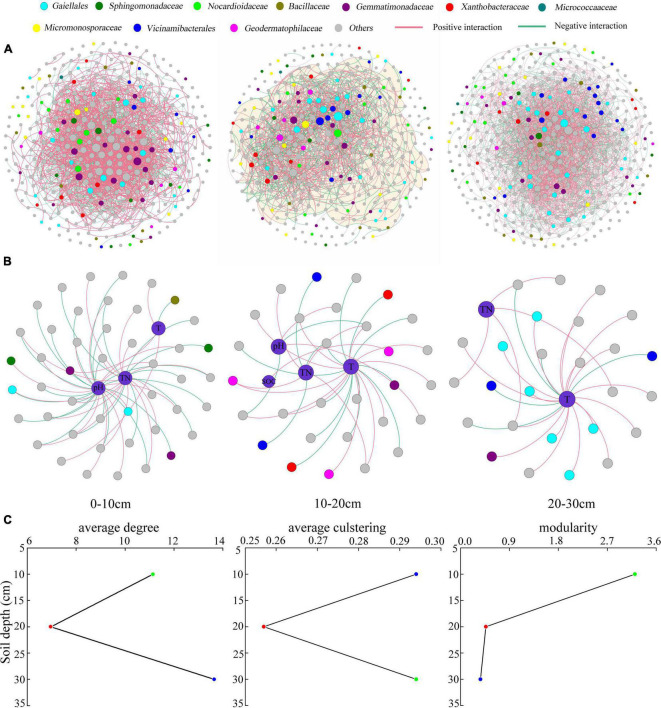
Taxon–taxon networks **(A)**, taxon–environment networks **(B)**, and statistics for taxon–taxon networks **(C)** in the three soil layers. In panels **(A,B)**, the connection indicates a strong and significant (*P* < 0.01) correlation; the nodes represent unique sequences in the datasets; the size of each node is proportional to the relative abundance. In panel **(C)**, the green, red, and blue circles in each subplot represent the soil layers 0–10, 10–20, and 20–30 cm, respectively. TN, total nitrogen; T, tillage; SOC, soil organic matter.

The average degree and clustering coefficient first decreased and then increased with the soil depth, indicating that the taxon–taxon co-existing network was simple in the 10–20-cm soil layer and complex in the 20–30-cm soil layer; the taxons were more interconnected in the 20–30-cm soil layer (Figures 5A,C). The study also found that compared with the relatively isolated deeper soil layer, the 0–10-cm soil layer was more exposed to environmental interference, corresponding to higher modularity in the soil bacterial community, which was vital for maintaining the stability of the soil bacterial composition (Figure 5C). TN and tillage method were the most important factors strongly associated with soil bacterial taxa in the three soil layers. However, pH was strongly associated with taxa in the 0–10- and 10–20-cm soil layers (Figure 5B).

## Discussion

### Effect of deep tillage combined with biochar application on soil physicochemical properties

Agricultural soil bulk density increased along with the soil depth. It was because that surface soil was easily disturbed by human activities and biological factors, such as the input of organic matter, intercropping of crop roots, and activities of soil animals, especially in an agricultural intensive area, which could loose soil and make the surface soil bulk density significantly lower than that of subsoil (Li et al., 2019). Numerous studies have shown that SOC was the main factor affecting the bulk density, and there was a significantly negative correlation between the two (Wang et al., 2014; Yang et al., 2016). Therefore, in our study, increasing SOC in the subsoil through deep tillage combined with biochar application reduced the bulk density.

Deep tillage inverted and completely mixed the surface and deeper soil and turned the surface nutrients into the deeper soil layer, improving the shallow plowing layer in the clay vertisol, and therefore, the soil nutrients in the 20–30-cm soil layer under deep tillage were higher than those under rotary tillage (Qi et al., 2021). Furthermore, biochar, with high pH and an alkalizing effect, increased the soil pH, consistent with the previous studies (Obia et al., 2015; Hussain et al., 2020). In addition, the biochar, with a highly porous structure and large surface area, had a strong adsorption effect on the nutrients. Due to its specific surface characteristics, it reduced the leaching of soil nutrient elements, maintained the soil fertility at a high level, and significantly affected the soil physicochemical properties (Gao and Deluca, 2020; Hussain et al., 2020; Pan et al., 2021). Meanwhile, the primary raw material of biochar is biomass, which contains various nutrients such as N and P. Consistent with several previous studies (Liu et al., 2017; Gao and Deluca, 2020), this study also showed that biochar application increased soil nutrients and productivity. It is worth noting that the soil pH of the CK treatments was significantly higher than that of the NPK treatments in all periods, especially in the 2019 winter wheat maturity stage, and the soil pH of the CK treatments was significantly higher than that of the NPK treatment by about 1 pH unit, probably due to rainfall. The low rainfall during the sampling period led to poor mobility and a high accumulation of base cations in the surface soil (Tuo et al., 2020); therefore, the soil pH treated by CK treatment was high.

Further, deep tillage combined with biochar application lowered the soil bulk density, while it significantly increased the soil AP, SOC, TN, and NO_3_^–^–N content of the 20–30-cm soil layer, similar to the previous reports in various soil texture types (Gao and Deluca, 2020; Hu et al., 2021). Although the soil physicochemical properties were different, there interactions were displayed. For example, high SOC content could lower the soil bulk density by changing the soil texture and increase N and P contents in the soil by promoting N and P biochemical cycles (Huang et al., 2005; Adhikari and Bhattacharyya, 2015). In this study, the soil pH in the 20–30-cm soil layer was much higher than that in the 0–10-cm soil layer, which showed serious acidification occurred on the soil surface. Because the fertilizer was applied to the topsoil and a little fertilizer seeped into the deeper layers by rainwater and floodwater (Savin et al., 2021), the soil nutrient content in the 20–30-cm soil layer was lower than those in the topsoil. Thus, deep tillage and biochar application not only improved soil chemical nutrients, but also alleviated acidification of the vertisols.

### Effects of soil physical properties on the structure of soil bacterial community

Deep tillage and biochar indirectly affect bacterial abundance *via* affecting the flow of water and air, which are the main factors needed for microbial growth (Abujabhah et al., 2016; Wang et al., 2020). The soil bacterial community composition varies with soil depth, and oxygen in different soil depths causes this difference. For example, the abundance of aerobic bacteria, such as *Sphingomonadaceae* and *Bacillaceae*, was higher in the topsoil than that in the subsoil. On the contrary, the abundance of *Methylomirabilota*, a type of anaerobic bacteria, was significantly higher in the subsoil than that in the topsoil. Consistent with the results of previous related studies, it was observed in the current study that deep tillage promoted the activation of aerobic microorganisms, such as *Gaiellale* and *Bacillaceae*, by improving the soil permeability and decreasing the soil bulk density (Wang et al., 2020, 2021).

In this study, although the soil bacterial communities varied in the different layers, no significant change in the dominant species of the community structure was observed. The similarity in the vertical distribution of the dominant soil bacterial species might be due to their strong diffusion effect, distribution range, or strong adaptability to the environmental changes in these bacterial species, which easily resulted in the random and extensive distribution characteristics (He and Ge, 2008). Besides, biochar application increased the abundances of *Sphingomonadaceae* and *Micrococcaceae*, mainly due to an increase in the soil bacterial community and activity (Fang et al., 2020; Sun et al., 2020). The decreased soil bulk density under deep tillage and with biochar additions implied the increased soil air permeability. As reported in several previous studies, due to the porous structure and large surface area (Hussain et al., 2020; Pan et al., 2021), the biochar application created a suitable living environment for soil bacteria to grow and breed by improving the soil porosity (Abujabhah et al., 2016; Hussain et al., 2020). In addition, biochar could provide safe niches for the soil bacteria to protect against predation by the protozoa (Azeem et al., 2021). Moisture content is the main factor affecting the biological activity; however, no significant effects of soil moisture on soil bacterial composition or diversity were detected in this study, probably due to the subtle difference between soil layers in the clay soils.

### Effects of soil chemical properties on the bacterial community

Previous studies have demonstrated that the soil chemical properties are the important factors affecting the structure of soil bacterial communities (Wei et al., 2019; Sun et al., 2020). This study’s taxon–environment network analysis showed that the soil pH, TN, and SOC were strongly correlated with bacterial taxa in the 0–30-cm soil layers (Figure 5B), consistent with the studies of Qiu et al. (2020) and Wan et al. (2020). We found that pH and SOC significantly affected the bacterial community only in the 0–10- and 10–20-cm soil layers and were the major factors driving the bacterial community. The soil layers in 0–10 and 10–20 cm depth had an acidic environment that bacteria preferred. Meanwhile, SOC, a vital source in the microbially driven carbon cycle, was mainly distributed in the topsoil, and therefore, the changes in pH and SOC are more associated with the soil bacteria (Puissant et al., 2015; Cao et al., 2016). The studies have identified the soil pH and tillage as important factors determining the distribution pattern of microorganisms (Xia et al., 2020; Shanmugam et al., 2021). The soil pH was an important factor that influenced the structure and diversity of soil metabolism and bacterial community (Siles and Margesin, 2016; Cheng et al., 2020). Sun et al. (2015) found that the structure of the soil bacterial community in soils with similar pH had similar characteristics, consistent with the conclusion of this study.

Different tillage operations lead to the breaking up and incorporation of crop residues into different soil depths and accelerate the decomposition and mineralization processes as the microbial decomposition acts on the available nitrogen, carbon, and other nutrients (Le et al., 2019). Deep tillage provided the nutrients for soil microorganisms to grow by turning the last crop stalks into deeper soil, which impacted the activity and the abundance of soil microorganisms (Gregorutti and Caviglia, 2019; Lou et al., 2021). Thus, the bacterial community composition of the bottom soil also changed. On the contrary, tillage might have reduced the habitat heterogeneity, favoring few bacteria and helping them outgrow other competitors; this eventually changed the bacterial community (Shanmugam et al., 2021), by altering the chemical properties.

### Effects of soil physicochemical properties and bacterial community structure on crop yield

Soil physicochemical properties directly affect crop yield by influencing nutrient input or indirectly by altering soil microorganisms (Lei et al., 2019; Chen et al., 2020). Soil physicochemical properties such as N, P, and K were essential for crop growth and maintenance and improvement in soil quality, which resulted in high crop yield (Belay et al., 2002; Zhao et al., 2013; Eo and Park, 2016). Moreover, soil bacterial community composition was sensitive to variations in the environmental features such as soil properties, and these soil bacteria played a key role in determining crop yield (Berg, 2009; Vasconcellos et al., 2013; Sun et al., 2015). Deep tillage broke the soil obstacles and improved the aeration, which benefited crop root growth (Muche et al., 2017; Schneider et al., 2017). In this study, deep tillage in the vertisol soil increased the winter wheat yield significantly (*P* < 0.05) compared with the traditional rotary tillage. However, the increase in maize yield under deep tillage was insignificant, contradicting the yield increase under deep tillage (Schneider et al., 2017; Wang et al., 2021). This inconsistency may be due to the soil environment or seasonal climate differences. We also found that the soil NO_3_^–^–N content was significantly and positively associated with crop yield in the three soil layers (Table 6A). In acid soil, the soil pH of the 0–10- and 10–20-cm soil layers was positively associated with crop yield, while an opposite association was observed in the 20–30-cm soil layer. Pan et al. (2020) reported that low soil pH reduced N uptake and inhibited maize root in the topsoil, but a neutral pH in the subsoil promoted N absorption by the crop. Meanwhile, the NH_4_^+^–N and TN content of the 20–30-cm soil layer was negatively associated with the crop yield, probably because high N inhibited biological N fixation (Ding et al., 2020).

**TABLE 6:** (A) Correlation of soil properties, soil bacterial species richness, and grain yields (*N* = 5).

	Depth	pH	SWC	NH_4_^+^–N	NO_3_^–^–N	SOC	TN	AP	Species richness
Yield	0–10 cm	0.243	0.419	0.141	0.860*	0.043	0.535	0.454	0.069
	10–20 cm	0.099	0.490	0.216	0.855*	0.568	0.193	0.668	−0.046
	20–30 cm	−0.565	0.467	−0.549	0.808*	−0.001	−0.153	0.649	0.480

Significant effects (*P* < 0.05) are labeled with “*”.

Besides, few soil bacteria might have altered the effect on the soil nutrient availability (Zhang et al., 2020). Only the abundances of *Gaiellalea*, *Sphingomonadaceae*, and *Nocardioidaceae* were positively correlated with the crop yield (*r* = 0.866, *P* < 0.05; *r* = 0.883, *P* < 0.05; and *r* = 0.810, *P* < 0.05, respectively), while the abundance of *Vicinamibacterales* was negatively correlated with the crop yield (*r* = −0.945, *P* < 0.05) (Table 6B). In the soil, bacteria belonging to *Sphingomonadaceae* were noted for secreting acidic exopolysaccharides, and it could protect the rhizosphere and promote the rooting by biodegrading a variety of complex xenobiotics (Johnsen et al., 2000; Benson et al., 2004). This study found that deep tillage combined with biochar increased subsoil’s bacteria abundance and bacteria diversity indexes. As the bacterial abundance improved, it increased solubilization of AP. Increased uptake of AP might enhance root growth that ultimately enhanced the uptake of nutrients, eventually resulting in better yield (Siddiq et al., 2018; Azeem et al., 2021). Bacterial diversity increased the soil quality fertility and the soil fertility, which were important for nutrient cycling and improving the plant health. Thus, the soil physicochemical properties and bacteria had a significant effect on the crop yield. Meanwhile, deep tillage combined with biochar indirectly increased crop yield by improving soil physicochemical properties and altering the bacterial community.

**TABLE 6:** (B) Correlation of soil bacterial abundance at the family level and grain yields (*N* = 5).

Species	Yield	Species	Yield
*Gaiellalea*	0.866*	*Xanthobacteraceae*	0.158
*Sphingomonadaceae*	0.883*	*Micrococcaceae*	−0.666
*Nocardioidaceae*	0.810*	*Micromonosporaceae*	−0.190
*Bacillaceae*	0.704	*Vicinamibacterales*	−0.945*
*Gemmatimonadaceae*	−0.660	*Geodermatophilaceae*	0.403

Significant effects (*P* < 0.05) are labeled with “*”.

## Conclusion

This study found deep tillage combined with biochar application as a reliable agricultural practice for soils with high clay content, superficial topsoil, and soils with poor nutrient content. This agricultural practice broke the physical obstacles in the clay vertisols of the North China Plain, improving the clay vertisol texture and shallow plowing layer. At the same time, deep tillage combined with biochar application enhanced the nutrient content (NO_3_^–^–N, AP, SOC, and TN) in the subsoil and subsequently improved the crop yield. However, this study carried out deep tillage and sampling only at the 0–30-cm soil layer. Further studies involving long-term experiments are needed for a better understanding of the effects of deeper tillage at other soil depths to improve the vertisol. This study provides evidence for increasing grain production and improving the vertisol *via* deep tillage combined with biochar application.

## Data availability statement

The datasets presented in this study can be found in online repositories. The names of the repository/repositories and accession number(s) can be found below: https://www.ncbi.nlm.nih.gov/, PRJNA827575.

## Author contributions

PL designed the study. YH and WC performed the field experiments and conducted the fieldwork. WC, FL, and JX conducted the laboratory work. WC and PL analyzed the data. WC wrote the manuscript. All authors read and approved the final manuscript.

## Footnotes

1
http://drive5.com/uparse/

2
http://rdp.cme.msu.edu/
